# Access to and utilisation of GP services among Burmese migrants in London: a cross-sectional descriptive study

**DOI:** 10.1186/1472-6963-10-285

**Published:** 2010-10-12

**Authors:** Nyein Chan Aung, Bernd Rechel, Peter Odermatt

**Affiliations:** 1 Epidemiology & Public Health Department, Swiss Tropical and Public Health Institute, 4002 Basel, Switzerland; 2 University of Basel, Basel, Switzerland; 3 European Observatory on Health Systems and Policies, London School of Hygiene & Tropical Medicine, London WC1E 7HT, UK

## Abstract

**Background:**

An estimated 10,000 Burmese migrants are currently living in London. No studies have been conducted on their access to health services. Furthermore, most studies on migrants in the United Kingdom (UK) have been conducted at the point of service provision, carrying the risk of selection bias. Our cross-sectional study explored access to and utilisation of General Practice (GP) services by Burmese migrants residing in London.

**Methods:**

We used a mixed-method approach: a quantitative survey using self-administered questionnaires was complemented by qualitative in-depth interviews for developing the questionnaire and triangulating the findings of the survey. Overall, 137 questionnaires were received (a response rate of 57%) and 11 in-depth interviews conducted. The main outcome variables of the study included GP registration, barriers towards registration, GP consultations, barriers towards consultations, and knowledge on entitlements to health care. Quantitative data were analysed using descriptive statistics, association tests, and a multivariate analysis using logistic regression. The qualitative information was analysed using content analysis.

**Results:**

The respondents were young, of roughly equal gender (51.5% female), well educated, and had a fair level of knowledge on health services in the UK. Although the GP registration rate was relatively high (80%, 109 out of 136), GP service utilisation during the last episode of illness, at 56.8% (54 out of 95), was low. The statistical analysis showed that age being younger than 35 years, lacking prior overseas experience, having an unstable immigration status, having a shorter duration of stay, and resorting to self-medication were the main barriers hindering Burmese migrants from accessing primary health care services. These findings were corroborated by the in-depth interviews.

**Conclusions:**

Our study found that having formal access to primary health care was not sufficient to ensure GP registration and health care utilisation. Some respondents faced difficulties in registering with GP practices. Many of those who have registered prefer to forego GP services in favour of self-medication, partly due to long waiting times and language barriers. To ensure that migrants enjoy the health services they need and to which they are entitled, more proactive steps are required, including those that make health services culturally responsive.

## Background

Migration has a major impact on physical, mental and social dimensions of health, as well as on access to and utilisation of health services by migrant and host populations. Burma (Union of Myanmar) is one of the least developed countries worldwide. Its human development index ranked 138 of 182 assessed countries [1]. An estimated population of 48 million lived in the country in 2009 [2]. The country has suffered the consequences of a repressive military dictatorship since 1962, which have included economic hardship and political turmoil, compounded by armed conflict in the border areas. By 2005, at least 3 million people had emigrated, most of whom to neighbouring countries in Southeast Asia [3]. However, places of destination also included high-income countries, such as the United States, Australia, Canada, United Kingdom, Ireland, Finland, the Netherlands, New Zealand, Norway and Sweden [4]. Research conducted in Thailand, where the vast majority of Burmese emigrants are residing, revealed that even in a country with a similar cultural background, Burmese migrants were facing a number of barriers to accessing health services [5,6]. To our knowledge, no research has so far been undertaken on access of Burmese migrants to health services in European countries, including the UK. Although the health care system in the UK is based on the dual principle of universality and fairness, the April 2004 amendments to the Department of Health charging regulations for overseas visitors tightened the conditions of entitlement to free access to NHS health services by migrants [7-9]. Although a court ruling in April 2008 has entitled most refused asylum seekers to free NHS health care (both primary and secondary), other undocumented migrants can access primary health care only at the discretion of General Practitioners [10]. While many other migrant groups apart from Burmese migrants have been covered by research, most studies in the UK were conducted at the point of service delivery, leading to selection bias [11-13].

By September 2008, the Office for National Statistics estimated that there were 3,000 Burmese-born people living in London (error margin +/- 3,000). The 2001 census revealed 3,534 Burmese-born migrants in 32 London boroughs at the time of the census, while the Department of Work and Pension reported that 1,990 Burmese migrants had undergone national insurance number registration during the period from 2003 to 2008.

Our study aimed to assess knowledge of Burmese migrants on health services in Greater London, the current level of access to and utilisation of General Practice (GP) services, barriers or obstacles encountered during GP registration and when consulting GPs, and socio-demographic disparities in access to health care within Burmese migrants.

## Methods

### Study design

We applied a mixed-method approach of quantitative and qualitative techniques. Six in-depth interviews before the quantitative survey helped to estimate the size of the Burmese migrant population in London, identify volunteers for questionnaire distribution, and prepare, pre-test and finalize the questionnaire. Five in-depth interviews followed the survey to triangulate and validate the quantitative findings and provide more insights into some of the sensitive areas covered. The variables of interest included explanatory factors, such as socio-economic and demographic indicators, and outcome variables, such as knowledge on entitlements to health services, access to GP services, GP service utilisation during last episode of illness, and barriers in accessing and utilising GP services.

### Study population and sample size

The sampling frame comprised Burmese migrants residing in Greater London. Although official figures cited earlier were much lower [14], based on the in-depth interviews with Burmese key informants, we estimated that there were about 10,000 Burmese migrants in Greater London in 2009. Epi Info™ (Version 6) was used to calculate the required sample size of the survey. The result was that a representative sample of 162 respondents was needed, based on an estimate of the main outcome variable of GP registration of 69% [15], a 10% error margin, a 95% confidence interval, and taking consideration of the effect of the snowball sampling method [16]. We decided to distribute 231 self-administered questionnaires, in the expectation of a response rate of 70%, when using measures to maximise response [17]. The selection criteria of the survey required that respondents were Burmese migrants aged 15-60 years and residing in Greater London. An upper age limit was used in view of the higher utilisation of health services among older people. For the in-depth interviews, we recruited Burmese migrants from different socio-economic clusters who had lived in London for more than 5 years and in at least 3 different areas of London, to ensure that they had a broad knowledge about the Burmese community in London.

### Study tools

The self-administered questionnaire and topic guideline for the in-depth interviews were developed by the bilingual lead author, based on a Europe-wide study on undocumented migrants' access to health care [18]. The questionnaire covered socio-demographic characteristics, knowledge on rights and entitlements to health care services, as well as access to and utilisation of health care in the UK. Ten questions were used to determine awareness of entitlements: five on GP registration of migrants with different immigration status, four on costs of prescribed medication, accident and emergency services, inpatient care, and family planning, and a question on legal abortion (Additional file 1). The questionnaire was translated into Burmese, pre-tested and validated using in-depth interviews.

### Data collection

Altogether 240 self-administered questionnaires with pre-stamped return envelopes addressed to the lead investigator were distributed through 25 volunteers from different geographical areas of London who were identified and recruited through the respondents of the in-depth interviews. Using a snowball approach, each volunteer distributed about 10 questionnaires in his/her area. A total of 137 questionnaires were posted back during the survey period (11 July to 9 September 2009), resulting in a response rate of 57%. Qualitative in-depth interviews were conducted to prepare the quantitative survey (6 pre-survey in-depth interviews, Additional file 2) and to triangulate the quantitative findings (5 post-survey in-depth interviews, Additional file 3). Informed consent was obtained from all participants: written consent for the questionnaire survey, using a question at the beginning of the questionnaire following the information sheet (Additional file 4), and verbal consent for the in-depth interviews. Ethical clearance was obtained from the ethics committee of the London School of Hygiene & Tropical Medicine (18 June 2009).

### Analysis

The quantitative data were double entered and analysed using SPSS, version 17.0. A descriptive analysis was run on outcome and independent variables. Linear associations were sought between continuous explanatory variables (namely: age, working hours per week, income, duration of stay in the UK, duration of stay in foreign countries, number of foreign countries ever resided in, total knowledge score on entitlements to and costs of health care in England, time lapse from first arrival to the UK to GP registration attempt and waiting time for GP consultation) and GP registration, using Pearson's Correlation test. The independent variables, such as age, education level, experience of living in other foreign countries, immigration status, National Insurance Number registration status, communication skills in English (speaking, listening and describing illness) were re-coded into binary variables and their associations with the main outcome variable (GP registration) were tested using chi square. Multivariate analysis was conducted to explore the strength of association between GP registration and explanatory variables (p value less than 0.07) during bivariate analysis. Content analysis was performed to analyse the in-depth interviews following the survey, using audio records and field notes.

## Results

### Socio-demographic characteristics

The respondents had a mean age of 31.6 years (SD = 8.95, n = 137) and a male-to-female ratio of 0.93. The majority of the respondents (70%) had at least university-level education and less than 6% were not high school graduates. More than 85% of respondents reported having at least a fair level of English (listening, speaking and describing illness) on a 5-point scale, ranging from very poor to poor, fair, well, and very well.

**Figure 1:**
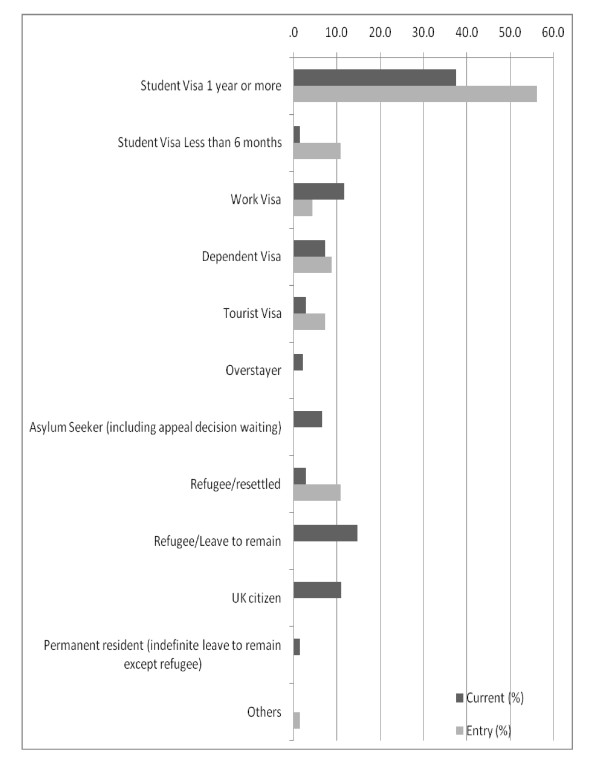
**Entry (n = 137) and current immigration status (n = 136) among respondents (September 2009)**.

Of all respondents, 85.4% (117 out of 137) reported to have a National Insurance Number and 80.3% (110 out of 137) reported that they were working. Regarding the current visa status, 37.5% of respondents were students who had stayed in the UK for one year or more, followed by respondents with refugee status or leave to remain (14.7%), work visa (11.8%), UK citizens (11%) and others (Figure 1).

### Knowledge

**Figure 2:**
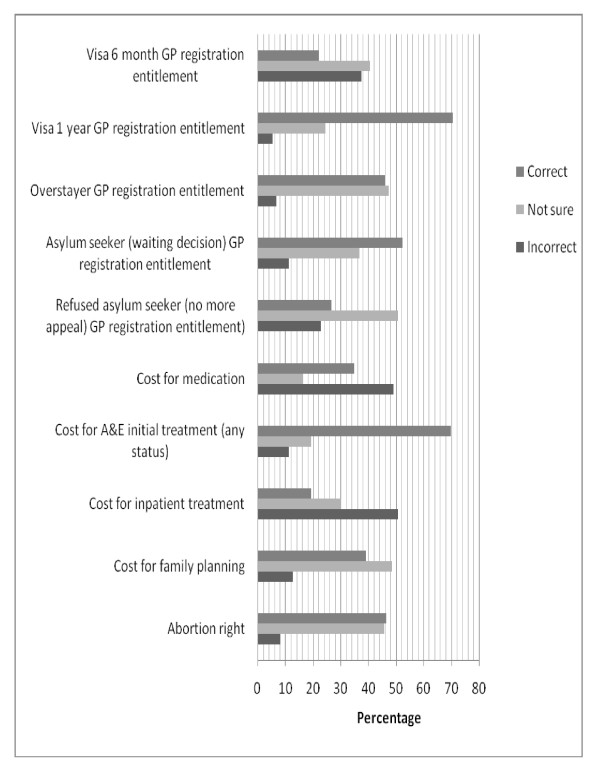
**Percentage of correct, not sure and incorrect answers on knowledge questions (September 2009)**.

Around 50% of respondents had correct knowledge on GP registration entitlement of asylum seekers, overstayers and students having 1 year visa. Only 22.0% gave the correct answer to a question regarding GP registration entitlement of migrants holding a 6-month student visa (Figure 2).

Knowledge on health care entitlements of asylum seekers was negatively associated with GP registration, as those who were registered had a lower knowledge on health care entitlements of asylum seekers than the unregistered population, but the association was only marginally significant (OR = 0.43, 95% CI = 0.18-1.01). The good knowledge on registration entitlements can be illustrated by the quote of one participant in the in-depth interviews: "*Yes, I know I am entitled to get registered with a GP, but I have been waiting for 2 months, as I need someone to accompany me to the GP surgery*".

### GP registration

The current GP registration rate was taken as an important indicator of migrants' access to primary health care services. However, while 84% of respondents had reportedly tried to register with a GP, only 79% (n = 136, 95% CI = 76-83%) of respondents were registered with a GP at the time of the survey. From the 5 respondents who tried to register with a GP but failed to do so, 4 did not have the necessary documentation and one could not overcome the language barrier when trying to communicate with the staff of the GP surgery.

The association of socio-demographic variables with GP registration status is shown in Table 1. The following variables (binary) showed a significant association and favoured GP registration: age (35 years and above: OR = 3.16, 95% CI = 1.12-8.93), education (graduate and postgraduate: OR = 2.40, 95% CI = 0.95-6.06), immigration status (having a stable immigration status: OR = 9.81, 95% CI 3.18-30.28), legal work status (having a National Insurance Number: OR = 10.82, 95% CI = 3.71-31.58), overseas experience (having been in a foreign country other than the UK for more than one month: OR = 6.35, 95% CI = 1.42-28.41) and communication skills in English (being able to speak, listen and describe an illness well or very well).

**Table 1:** Effect of socio-demographic factors on GP registration, showing odd ratios and significance level

Socio-demographic		GP registration Number (%)		Odd ratios	95% Confident
factors		Yes	No		Interval
Age	35 and above	44 (40.7%)	5 (17.9%)	3.16	1.12-8.93
			
	Under 35	64 (59.3%)	23 (82.1%)	1	

Education level	Graduate and postgraduate	76 (72.4%)	12 (52.2%)	2.40	0.95-6.06
			
	Under graduate	29 (27.6%)	11 (47.8%)	1	

Been in a foreign country for more	Yes	36 (34.6%)	2 (7.7%)	6.35	1.42-28.42
			
than a month apart from UK	No	68 (65.4%)	24 (92.3%)	1	

Immigration status	Others	67 (62.0%)	4 (14.3%)	9.81	3.18-30.28
			
	Asylum seekers, overstayers and students (6 month)	41 (37.9%)	24 (85.7%)	1	

National Insurance Number	Have	101 (93.5%)	16 (57.1%)	10.82	3.71-31.58
			
	Do not have	7 (6.5%)	12 (42.9%)	1	

English listening Skill	Understand very well and well	60 (55.6%)	9 (32.1%)	2.64	1.10-6.36
			
	Understand fairly and poorly	48 (44.4%)	19 (67.9%)	1	

English speaking skill (general)	Speak very well and well	54 (50.0%)	7 (25.0%)	3.0	1.12-7.64
			
	Speak fairly and poorly	54 (50.0%)	21 (75.0%)	1	

English speaking Skill (regarding	Can express illness very well and well	42 (39.6%)	5 (17.9%)	3.02	1.06-8.56
			
illness)	Can express illness fairly and poorly	64 (60.4%)	23 (82.1%)	1	

Age (n = 136, r = 0.307, p < 0.001), weekly net income (n = 93, r = 0.276, p = 0.007), duration of stay in the UK (n = 134, r = 0.344, p < 0.001) and duration of stay in foreign countries apart from the UK (n = 127, r = 0.205, p = 0.021) had a significant positive correlation with GP registration status.

Multivariate logistic regression showed that "having foreign experience", "having stable immigration status" and "having a National Insurance Number" continued to have a statistically significant association with GP registration after all the others factors were adjusted (Table 2).

**Table 2:** Multivariate analysis and adjusted odd ratios among the factors influencing GP registration

**Currently registered with a GP**^**a**^	Adjusted odd ratio (OR)	95% Confidence Interval for Adjusted Odd Ratio	
		
		Lower Bound	Upper Bound
Age younger than 35 years (versus 35 and older)	0.496	0.112	2.184
Having stable immigration status (versus not having)	5.543	1.219	25.200
Having National Insurance Number (versus not having)	9.974	1.937	51.361
Not being graduated (versus graduated)	0.702	0.202	2.439
Having foreign experience (versus not having)	9.592	0.984	93.523
English listening skill: Understand very well and well (versus below)	0.336	0.040	2.785
English speaking skill: very well and well (versus below)	3.840	0.317	46.557
Ability to describe illness: very well and well (versus below)	1.248	0.150	10.370

a. The reference category is: No, haven't registered with GP

**Figure 3:**
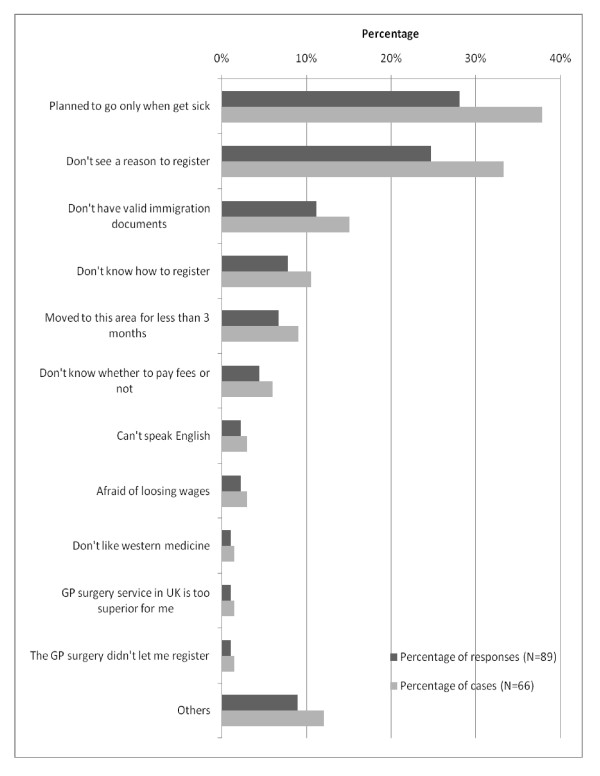
**Reasons hindering GP registration (multiple-response question) for case-wise (n = 66) and response-wise (n = 89), (September 2009)**.

Among those respondents who reported reasons that deterred them from GP registration, "planned to go only when get sick" (37.9%) and "don't see a reason to register" (33.3%) were the most prevalent reasons (Figure 3).

### GP consultation during last episode of illness

**Figure 4:**
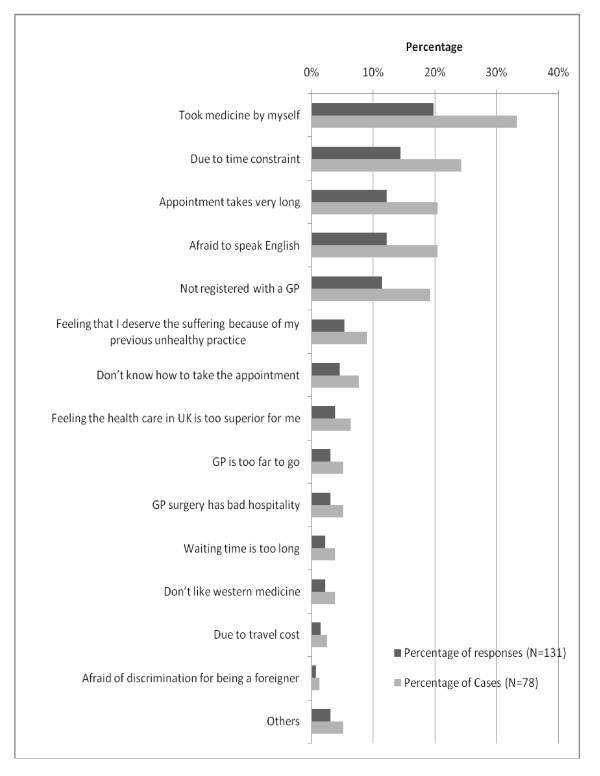
**Reasons hindering GP consultation (multiple-response question), for case-wise (n = 78) and response-wise (n = 131), (September 2009)**.

Only 56.8% (N = 95) of respondents went to their GP clinic during their last episode of illness. Of all respondents, 21.8% (29 out of 133) did not indicate any reason deterring them from GP consultations. Among those who reported recent episodes of illness and faced barriers in utilizing GP services, "self-medication" was the most prevalent reason, deterring 33.3% (26 out of 78) of respondents from GP consultations, followed by time constraint (24.4%), prolonged waiting times for appointments (20.5%), being afraid to speak English (20.5%), not being registered with a GP (19.5%), and other different reasons (respondents could choose more than one answer; Figure 4).

**Figure 5:**
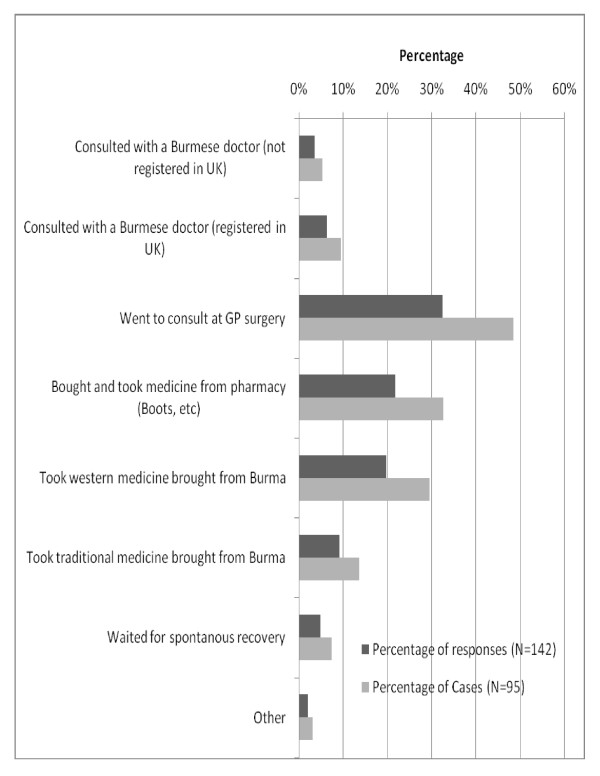
**Actions taken during last episode of illness (multiple response question), for case-wise (n = 95) and response-wise (n = 142), (September 2009)**.

When exploring the actions during the last reported episode of illness, GP consultation and self-medication were found to be the most prevalent actions (Figure 5). Regarding self-medication practice, an in-depth interview respondent reflected on the medical supplies that he had brought with him from Burma. "*Well, I brought medicine since I left Burma: both Burmese traditional medicine and western medicine. I have antibiotics like Ampicillin, Amoxycillin and Tetracycline. I even have anti-TB and anti-malaria drugs. I also have a medical person in close relative (overseas) so whenever I was not well, I rang them and took advice through telephone consultation."*

## Discussion

Our study found a relatively high GP registration rate (80%, 109 out of 136), but a low rate of utilizing GP services during the last episode of illness (56.8%, 54 out of 95). The statistical analysis showed that age being younger than 35, lacking prior overseas experience, having an unstable immigration status, having a shorter duration of stay, and resorting to self-medication were the main barriers hindering Burmese migrants from accessing primary health care services.

When drawing conclusions from our findings, however, several limitations need to be borne in mind. While we aimed to obtain a representative sample of Burmese migrants in London, several intrinsic difficulties mean that the findings cannot easily be generalised to all Burmese migrants in London, nor to other groups of migrants. Due to the lack of data on the migration status of the population, we had to rely on an estimated size of the Burmese migrant population in London and on a snowball sampling method to recruit respondents. This inevitably introduced a bias that is difficult to quantify. When considering the socio-demographic profile of respondents, it is striking that they were younger (95% under the age of 45) and had migrated more recently (92.6% have stayed in London for less than 10 years) than international migrants covered in other studies [8,19,20]. Furthermore, a high proportion of respondents (95%) were high school or university graduates, which is similar to the level of education of Nepalese migrants in the United Kingdom [20], and 85% reported to have at least a fair level of English speaking (both general and when describing illness) and listening skills.

A comparatively low response rate (57%) and unanswered questions present additional limitations to our study. It should also be noted that some of the questions (related to immigration status and income) were of a very sensitive nature, which may have influenced the results and the participation rate. In view of these limitations, we took several steps to increase the validity of our findings. We used key informants at the community level for estimating the size of the Burmese migrant population and to distribute the questionnaires across different socio-economic clusters, and adjusted sample size for the design effect of snowball sampling. The triangulation of data through the use of both quantitative and qualitative methods further allowed to explore issues in more depth and to double-check findings and interpretations.

Our study revealed several key issues with regard to the utilisation of primary health care by Burmese migrants in London. The GP registration rate of our respondents (80%) was higher than that of international migrants who had attended the accident and emergency services at an inner London hospital (68.8%) [15]. As discussed in the previous section, the factors which showed significant association with GP registration in bivariate analysis were: age, education level, experience and duration of stay in other foreign countries, immigration status, National Insurance Number registration status, English language proficiency, income, and duration of stay in the UK. These findings are consistent with some of the observations made in a study on international migrants admitted to an infectious disease department of an inner city London hospital, which found that non-registration with GPs was strongly associated with being overseas born, being a refugee or asylum seeker, having arrived in the UK recently, and not having English as a first language [21].

In our study, socio-demographic factors seem to have a stronger effect on GP registration status than the level of knowledge on health care entitlements. Surprisingly, having knowledge of health care services and GP registration did not show significant association with GP registration, except for the knowledge on health care entitlements of asylum seekers, where the registered population had less knowledge than the unregistered population. It might be concluded that the GP registration status of Burmese migrants is independent of their knowledge on health care entitlements. However, to reach such a conclusion, a thorough sub-group analysis for each immigration status would be necessary. This was not possible within the framework of our study, as the numbers of respondents in each subgroup were insufficient for this type of analysis. Indeed, other studies suggest that insufficient knowledge of entitlements was a main barrier to health care utilization [22].

With regard to delayed GP registration, "planned to go only when get sick" (28%, N = 131) and "don't see a reason to register" (25%, N = 131) were the most prevalent reasons. This might reflect the experience of respondents back in Burma, where patients do not need to be registered with GPs, but instead seek treatment directly from hospitals or private clinics in case of illness. This interpretation of the quantitative findings was confirmed in the qualitative interviews, where a respondent pointed to "*the lack of prior experience with a GP system in Burma*" as a reason causing delayed GP registration. Cultural factors might also be at play. A study examining the uptake of antiretroviral treatment among African migrants living with HIV/AIDS in the UK found that: "health is only a priority when one is unwell; otherwise issues around immigration, housing, employment, and childcare take precedence" [22].

Only 21.8% (N = 133) of respondents in our study reported that there were no reasons for not utilizing GP services. The five leading reasons for not consulting their GPs, reported by 10% or more of respondents, were: self-medication, time constraint, afraid to speak English, not having GP registration, and prolonged waiting time for appointments. The utilisation of GP services at the last episode of illness in our study (56.8%, N = 95) was far below the GP utilisation rate of international migrants found in a survey conducted in 2005 by Médecins du Monde in 9 European countries including the UK, indicating a rate of 76% [18]. However, the study by Médecins du Monde also found that self-medication was the second most frequent action during the last episode of illness (49.5%), corroborating the high rate of self-medication found in our study. Our in-depth interviews revealed that the medicines brought from Burma included some that require prescriptions in the UK, such as antibiotics, anti-tuberculosis drugs and even anti-malaria medicines. In contrast, no prescription is needed to buy most of these medicines in Burma. Widespread self-medication of Burmese migrants has also been documented in other countries, mostly in Thailand [6,23,24].

## Conclusions

Our sample of respondents displayed several characteristics favouring access to health care services. They were comparatively young, well educated, had a fair level of communication skills in English, and a good knowledge of their entitlements to health care. Yet, despite an overall high rate of GP registration, some had faced problems in accessing GP services, and utilisation of GP services at the last episode of illness was relatively low. This might mean that utilisation may be even lower for other groups of migrants who face additional barriers to access, such as low levels of education, poor knowledge of English, or poor awareness of entitlements. More research is needed to establish utilisation rates of health services among other groups of migrants.

Our multivariate analysis showed that having a National Insurance Number, a stable immigration status and experience of living in other countries were strongly associated with being registered with a GP. Some of our respondents delayed GP registration, as no such registration is required in their country of origin. This means that health system factors need to be taken into account in efforts to improve health care utilisation by migrants. Our findings suggest that particular attention should be paid to migrants with an unstable immigration status and not presently engaged in formal employment, such as through the provision of information materials and telephone hotlines on health and immigration issues. Population mapping and community empowerment through a community based-development approach are other options for improving access of migrants to health services [25].

Another important finding of our study are high rates of self-medication, corroborating research on other migrant groups in Europe and on Burmese migrants in Southeast Asia [6,23,24,26]. This includes recourse to medicines that are only available with prescription in the UK, raising concerns about the appropriateness of the medicines used and potential public health risks, such as drug resistance arising from the use of antibiotics. More research is needed on current self-medication practices among migrants and ways of addressing them. This will have to include culturally sensitive ways of ensuring that migrants enjoy the health services they need and to which they are entitled.

## Competing interests

The authors declare that they have no competing interests.

## Authors' contributions

NCA planned and conducted the survey under the supervision and support of PO and BR, and carried out the data entry and analysis. PO and BR reviewed and revised the article drafted by NCA. All authors have read and approved the final manuscript.

## Pre-publication history

The pre-publication history for this paper can be accessed here:

http://www.biomedcentral.com/1472-6963/10/285/prepub

## Supplementary Material

Additional file 1 **Questionnaire for quantitative study**. Click here for file

Additional file 2 **Pre-survey in-depth interview record sheet**. Click here for file

Additional file 3 Post-survey in-depth interview guideline. Click here for file

Additional file 4 Information sheet for questionnaire survey. Click here for file

## References

[B1] Human Development Report http://hdr.undp.org/en/statistics/

[B2] The World Factbook: Burma https://www.cia.gov/library/publications/the-world-factbook/geos/bm.html

[B3] Asian Migration Centre Asian migrant yearbook 2005 2005 85

[B4] Resettlement of Myanmar refugees from Thailand camps hits 50,000 mark http://www.unhcr.org/4a4a178f9.html

[B5] Naing Using Theory-based Behaviour Change Communication For HIV-prevention among Burmese Fishermen in Phuiket Thailand: An Evaluation Study 2006 World Vision Foundation of Thailand 82

[B6] Saether ST Chawphrae U Zaw MM Keizer C Wolffers I Migrants' access to antiretroviral therapy in Thailand Tropical Medicine & International Health 2007 12 8 999 1008 10.1111/j.1365-3156.2007.01879.x 17697095

[B7] Anderson J Coming and going: some aspects of care for migrants with HIV in the UK J Infect 2008 57 1 11 15 10.1016/j.jinf.2008.05.002 18541306

[B8] Hargreaves S Holmes AH Saxena S Le Feuvre P Farah W Shafi G Chaudry J Khan H Friedland JS Charging systems for migrants in primary care: the experiences of family doctors in a high-migrant area of London J Travel Med 2008 15 1 13 18 10.1111/j.1708-8305.2007.00161.x 18217864

[B9] National Health Service, England (NHS) The National Health Service (Charges to overseas visitors) (Amendment) Regulation 2004 614 2004 NHS

[B10] Global Health Advocacy Project Yates T Access to Primary Healthcare for Refused Asylum Seekers and Undocumented Migrants Briefing on the regulations and recent legal developments: 08/11/13 2008 2008

[B11] Lindert J Schouler-Ocak M Heinz A Priebe S Mental health, health care utilisation of migrants in Europe Eur Psychiatry 2008 23 Suppl 1 14 20 10.1016/S0924-9338(08)70057-9 18371575

[B12] Mayor S Report on health of migrants to UK shows high risk of TB and HIV BMJ 2006 333 7578 1088 10.1136/bmj.333.7578.1088-b 17124210 PMC1661712

[B13] Palmer D Ward K 'Lost': listening to the voices and mental health needs of forced migrants in London Med Confl Surviv 2007 23 3 198 212 10.1080/13623690701417345 17822063

[B14] Data Management and Analysis Group Finella G London borough residents by country of birth; An analysis of 2001 Census data DMAG Briefing 2006/4 2006 London: Greater London Authority

[B15] Hargreaves S Friedland JS Gothard P Saxena S Millington H Eliahoo J Le Feuvre P Holmes A Impact on and use of health services by international migrants: questionnaire survey of inner city London A&E attenders BMC Health Serv Res 2006 6 153 10.1186/1472-6963-6-153 17134491 PMC1698917

[B16] Salganik MJ Variance estimation, design effects, and sample size calculations for respondent-driven sampling J Urban Health 2006 83 6 Suppl i98 112 10.1007/s11524-006-9106-x 16937083 PMC1705515

[B17] Dillman DA Mail and Internet Surveys; The Tailored Design Method 2007 Second John Wiley & Sons, Inc

[B18] Médecins du Monde Michel Degueldre, Teresa Gonzalez, Françoise Jeanson, Eleftheria Parthenopoulou European survey on undocumented migrant's access to health care 2006 Médecins du Monde

[B19] Data Management and Analysis Group Spence L A Profile of Londoners by Country of Birth; Estimates from the 2006 Annual Population Survey DMAG Briefing 2008 London: Greater London Authority

[B20] Adhikary P Simkhada P van Teijlingen E Raja A Health and lifestyle of Nepalese migrants in the UK BMC International Health and Human Rights 2008 8 1 6 10.1186/1472-698X-8-6 18500980 PMC2432045

[B21] Cooke G Hargreaves S Natkunarajah J Sandhu G Dhasmana D Eliahoo J Holmes A Friedland JS Impact on and use of an inner-city London Infectious Diseases Department by international migrants: a questionnaire survey BMC Health Serv Res 2007 7 113 10.1186/1472-6963-7-113 17659074 PMC1940251

[B22] Burns FM Imrie JY Nazroo J Johnson AM Fenton KA Why the(y) wait? Key informant understandings of factors contributing to late presentation and poor utilization of HIV health and social care services by African migrants in Britain AIDS Care: Psychological and Socio-medical Aspects of AIDS/HIV 2007 19 1 102 108 10.1080/09540120600908440 17129864

[B23] Entz A Prachuabmoh V van Griensven F Soskolne V STD history, self treatment, and healthcare behaviours among fishermen in the Gulf of Thailand and the Andaman Sea Sex Transm Infect 2001 77 6 436 440 10.1136/sti.77.6.436 11714943 PMC1744414

[B24] Suwanvanichkij V Displacement and disease: The Shan exodus and infectious disease implications for Thailand Confl Health 2008 2 4 10.1186/1752-1505-2-4 18341695 PMC2324075

[B25] Jayaweera H Health and access to health care of migrants in the UK Better Health Briefing 2010 19

[B26] Jimenez Rubio D Hernandez Quevedo C Differences in self-medication in the adult population in Spain according to country of origin Gac Sanit 2010 24 2 116.e1 8 10.1016/j.gaceta.2009.09.007 19931220

