# Supplementary material for: Access to and utilisation of GP services among Burmese migrants in London: a cross-sectional descriptive study
Source: BMC Health Serv Res. 2010 Oct 12;10:285. doi: 10.1186/1472-6963-10-285 (PMC2970605; doi:10.1186/1472-6963-10-285)
Supplement: Additional file 1 — Questionnaire for quantitative study. [file 1472-6963-10-285-S1.DOCX]

# Questionnaire for Quantitative survey

**I. Socio-demographic Information**

| **No.** | **Questionnaires** | **Answers and Codes** | | |
| --- | --- | --- | --- | --- |
| Please answer all the questions in order. For the questions that you have to choose one or more choices, please tick the box **()**, next to the question like this (🗹)**.**If you like to change the answer question which you have answered, please fill the answer box like this **■** and choose a new answer by checking another box. | | | |  |
| Q100 | Do you agree to take part in questionnaire survey? | 1. Yes …………..….….…..……..……..……..…….….…  2. No……………......……..……..……..….……..……..… |  | |
| Q101 | What is your sex?  ***(Choose only one answer)*** | 1. Male …………..….….…..……..……..……..……..…  2. Female ………......……..……..……..….……..……..… |  | |
| Q102 | Please choose your religion by ticking the relevant box.  ***(Choose only one answer)*** | 1. Buddhist…………..……..……..……..……..……..…  2. Christian………......……..……..……..……..……..…  3. Islam……………...……..……..……..……..……..……  4. Hindi…….………...……...……...……...……...……...  5. Others……………...……...……...……...……...……...  Please mention________________________________  ____________________________________________ |  | |
| Q103 | Please choose your ethnic by ticking the relevant box.  ***(Choose only one answer)*** | 1. Bamar ..………….….….….….….….….….….….…..  2. Kayin…………......……………………………………  3. Mon. ….………...……………………………………  4. Kachin …………...………………………………  5. Chin …...………...………………………………  6. Others……………...……...……...……...……...……...  Please mention ­­­­­­­­­­­­­­­­­­­­­­_________________________________ ________________________­­­­­­­­­­­­­­­____________________ |  | |
| Q104 | What was your age on your last birthday?  ***(Please write your age on last birthday)*** | ____ years | | |
| Q105 | What is the post code of the area you are currently living? | \|  \|  \|  \|  \|  \|  \|  \| \| --- \| --- \| --- \| --- \| --- \| --- \| --- \| | | |
| Q106 | What is the highest level of education you have completed? | Highest level of education completed __________ | | |
| Q107 | To what level, do you think, you could understand when you hear a native speaking English?  ***(Choose only one answer)*** | 1. Very well …………..……..……..……..……..……..…  2. Well ………......……..……..……..……..……..........…  3. Fair ……………...……..……..……..……..……..……  4. Poor…….………...……...……...……...……...……...  5. Very poor………...……...……...……...……...……... |  | |
| Q108 | To what level, do you think, you could speak English?  ***(Choose only one answer)*** | 1. Very well …………..……..……..……..……..……..…  2. Well ………......……..……..……..……..……..........…  3. Fair ……………...……..……..……..……..……..……  4. Poor…….………...……...……...……...……...……...  5. Very poor………...……...……...……...……...……... |  | |
| Q109 | What is your marital status?  ***(Choose only one answer)*** | 1. Single …………..……..…….........……..……..……..…  2. De-facto ......……..……..……..………..……..........…  3. Married ……...……..……..……..……..……..………  4. Separated .………...……...……...……...……...…….....  5. Divorced ………...……...……...……...……...…….....  6. Widow/er ………...……...……...……...……...……...... |  | |
| Q110 | Are you sharing a house with any one? | 1. Not sharing a house .…..……..……..……..…….….…  2. Sharing a house....……..……..……..….……..……..… |  | |
| Q111 | If you are sharing a house, who are you sharing with?  ***(can choose more than one answer)*** | 1. Not sharing a house …..…….........……..……..……..…  2. Boy friend/girl friend/Spouse ……..………..……..........  3. Parents /children/siblings …..……..……….…...………  4. Friends.………...……...……...……...………....…….....  5. Other relatives…...……...……...……...……...…….....  6. Strangers ………...……...……...……...……...……...... |  | |
| Q112 | Do you have NINO/ National Insurance Number? | 1. Yes, I have…..….….…..……..……..……..…….….…  2. No, I don’t have ....……..……..……..….……..……..… |  | |
| Q113 | Are you working?  ***(If you are working, please give the average working hour per week)*** | 1. Working……..….….…..……..……..……..…….….…  2. Not working…......……..……..……..….……..……..…  If you are working, what is your average working hours per week: ________ hrs |  | |
| Q114 | How are you working, please choose one? | 1. Not working………..  2. Cash in hand……….  3. Using NINo……….. | | |
| Q115 | What is your weekly average income after tax? | \|  \|  \|  \|  \| GBP per week \| \| --- \| --- \| --- \| --- \| --- \| | | |
| Q116 | Please select any of the status which currently applies to you.  ***(You can choose more than one status if these apply)*** | 1. Student………….………..  2. Dependent…….………….  3. Living on benefit..………  4. Visitor...……..……………  5. Working…..…….....……... | | |

**II. Immigration status, duration of stay in UK and foreign experience**

| **No.** | **Questionnaires** | **Answers and Codes** | | |  |
| --- | --- | --- | --- | --- | --- |
| Q201 | How long have you lived in the UK? | ____Years ____months | | |  |
| Q202 | Have you ever lived in any foreign country for more than a month before coming to the UK?  ***(Choose only one answer)*** | 1. Haven’t lived .….….…..……..……..……..…….….…  2. Have lived .…......……..……..……..….……..……..…  If you have lived in foreign countries (except UK),  2.1 Number of countries _______  2.2 Total duration in foreign countries except UK  ____ year s____ months | |  | |
| Q203 | What was your immigration status, when you first entered UK?  ***(choose only one answer)*** | 1. Student Visa 6 months and less…………………..……  2. Student Visa 1 year or more …………………………..  3. Dependent Visa…………………………………………  4. Work Visa……………………………………………..  5. Refugee/resettled …….………………………..............  6. Tourist visa …………………………………………….  7. Others (please specify) ……………………………….  ………………………………………………………….. |  | |  |
| Q204 | What is your CURRENT immigration status?  ***(choose only one answer)*** | 1. Student Visa 6 months and less………………..……..  2. Student Visa 1 year or more ……………………...…..  3. Dependent Visa…………………………….…………  4. Work Visa……………………………………………...  5. Tourist visa …………………………………………..  6. Refugee/resettled …….…………………………….......  7. Overstay…………………………………………............  8. Asylum seeker (including those waiting for appeal decision)………………………………............................  9. Refugee/leave to remain ………………………….  10. Refused Asylum seeker (failed and have to be deported)……………………………………...............  11. Indefinite leave to remain (except refugee)….……….  12. UK citizen……………………………………............  13. Others (please specify) ………………………………  ………………………………………………………….. |  | |  |

**III. Knowledge on payment and the right to access health care**

| **No.** | **Questionnaires** | **Answers and Codes** | | |
| --- | --- | --- | --- | --- |
|  | **Please choose one of the given options (right, wrong or unsure) for the following conditions:** | **Right** | **Wrong** | **Not sure** |
| Q301  Q302  Q303  Q304  Q305  Q306  Q307  Q308  Q309  Q310 | *A migrant with a 6 month Visa* is entitled to be registered with a GP to get free consultations.  *A migrant holding a one year Visa* is entitled to be registered with a GP to get free consultations.  *A migrant overstaying his visa* is entitled to be registered with a GP to get free consultations.  *An asylum seeker with an application under consideration* is entitled to be registered with a GP to get free consultations.  *A failed asylum seeker* (no more right to appeal) is entitled to be registered with a GP to get free consultations.  *A person entitled for free GP consultation* is also entitled to get free medication.  *Anyone residing in UK (regardless of immigration status and stay)* is entitled to receive immediate treatment for emergency conditions at hospital’s Accident and Emergency department.  *Anyone residing in UK (regardless of immigration status and stay)* *who has a health condition necessary to be admitted to hospital* can get free treatment at the hospital.  Family planning service is freely available for anyone residing in the UK regardless of their immigration status.  Unwanted pregnancy can legally be terminated/ aborted in the UK before certain term of pregnancy. |  |  |  |

***IV. Current Health Care Utilisation***

| **No.** | **Questionnaires** | **Answers and Codes** | | |
| --- | --- | --- | --- | --- |
| Q401 | Have you ever tried/planned to register with a GP in the UK? | 1. Have tried to register …..……..……..……..…….….…  2. Haven’t tried to register..……..……..…....................... | |  |
| Q402 | How many months after your first arrival to the UK did you plan/try to register with a GP?  ***(Answer only one option)*** | 1. Haven’t tried to register at GP surgery..……..…….….…  2. Not remember…………..……..……..….......................  3. Duration (after first arrival to first attempt to register with a GP) _______ months | |  |
| Q403 | Who helped/advised you to get registered with a GP?  ***(You can choose more than one option if these apply)*** | 1. Boy friend/girl friend/Spouse….………………………  2. Parents/siblings/children………………………………  3. Friends…………………………………………………  4. Other relatives…………………………………………  5. Self…………………………………………………….  6. No one…...…………………………………………….  7. Others ………………………………………………… (please specify) ________________________________  _____________________________________________ | |  |
| Q404 | Did you have problems in registering with the GP at that time? | 1. Never tried to register ….……………………………...  2. Didn’t have problem ………………………………….  3. Had problem …………………………………………..  **If you had problem**, what kind of problem did you encounter, please choose:  2.1 Couldn’t communicate well …………………………  2.2 Didn’t have the document necessary…………………  2.3 Others ………………..……………………………… (please specify) ________________________________  ______________________________________________ | |  |
| Q405 | Could you register with GP at that time? | 1. Never tried to register ….……………………………...  2. Could register ……….………………………………….  3. Couldn’t register …..…………………………………..  **If Couldn’t**, please choose the reason:  2.1 Couldn’t communicate well …………………………  2.2 Didn’t have the document necessary…………………  2.3 Others ………………..……………………………… (please specify) ________________________________  ______________________________________________ | |  |
| Q406 | Are you currently registered with a GP in your area? | 1. Registered …..……..……..………………...…….….…  2. Not registered…………..……..……..…....................... | |  |
| Q407 | What is the name of surgery?  ***(Answer only one option)*** | 1. Not registered with a GP …..………………...…….….…  2. Don’t know the name…..……..……..….......................  3. If you know the name of surgery, please write down here _________________________ | |  |
| Q408 | Did you have a chance to choose your GP doctor?  ***(Answer only one option)*** | 1. Not registered with a GP …..………………...…….….…  2. Could choose GP…..……..……..…...............................  3. Couldn’t choose GP ……………………………........... | |  |
| Q409 | Regarding GP registration, could you please choose the reasons deterring/deterred you from GP registration  ***(You can choose more than one option if these apply)*** | 1. Didn’t see any reason to register …………….………  2. Decided to go and register only if I get sick …………  3. Don’t know how to register…………...……………...  4. Didn’t registered as living in current address for less than 3 months ………………………..........................  5. Don’t like taking western medicine …………………  6. Have a feeling that health care in UK is too superior for me...…..…..…..….…..…..…..…..…..…..…..……  7. Afraid of being discriminated as a Burmese …..…….  8. Not sure if I have to pay for the consultation or not …  9. Don’t have valid immigration document…………......  10. Can’t speak English to communicate at surgery……  11. Afraid of losing wages while doing registration...….  12. The GP didn’t let me register…...……………...…...  13. I am already registered and nothing had deterred me from GP registration ……………………………………  14. Others …………………………………………….…  (please specify) ……………………………………………………………………………………………………………………………………………………………………………… |  | |
| Q410 | Please give the diagnosis (if you have known from your GP) of the health problem you encountered last time in the UK. ***(Answer only one option)*** | 1. Never had had a health problem …………...…….….…  2. Don’t know exactly ……..……..…...............................  3. Please write the diagnosis of health problem, if you know: ____________________________ | |  |
| Q411 | Could you please choose the health problems you have last encountered during your stay in the UK?  ***(Answer only one option)*** | 1. Respiratory tract problems (coughing, sneezing, sore throat, etc)………...…………….…………….…………  2. Gastro-intestinal problems (abdominal pain, diarrhoea, vomiting, indigestion, gastritis etc) ….……...  3. Musculoskeletal problems (muscle ache/pain, bone & joint pain, injury etc) ….…………….…………….……  4. Obstetrics and gynaecology problems (pregnancy and abortion)…………… ……..……..……..……..……..…  5. Family planning ………………………………………  6. STI ………......………………………………………...  7. Urinary problems ……………………………………...  8. Mental health problems (depression, psychosis etc)…  9. Cardiovascular (heart disease, hypertension) …………  10. ***No health problem*** during my stay in UK …….…….  11. Others …………………………………………………  Please specify…………………………………………...  ………………………………………………………….. |  | |
| Q412 | Please rate the severity of the health problem you encountered last time in the UK.  ***(Answer only one option)*** | 1. Never had had a health problem …………...…….….…  2. Severe ……..……..……..…..…..…...............................  3. Moderate …..……..……..……..……..……..………….  4. Mild …..……..……..……..……..……..……..……..… | |  |
| Q413 | When you had health problem the last time, did you go to GP/clinic?  ***(Answer only one option)*** | 1. Never had had a health problem …………...…….….…  2. Went to GP ……..…..…..…............................................  3. Didn’t go to GP ….……..……..……..……..…………. | |  |
| Q414 | How long did you wait to get an appointment?  ***(Answer only one option)*** | 1. Never had had a health problem/never been to GP….…  2. Didn’t have to wait even a day....…..…..…......................  3. Number of days waited _________ | |  |
| Q415 | What is your level of satisfaction on GP’s consultation during your last visit?  ***(Choose only one option)*** | 1. Never have been to GP in UK …………...…….….…...  2. Fully satisfied …..……..…..…..….................................  3. Considerably satisfied...……..……..……..……………  4. Moderately satisfied ….....................................................  5. A little satisfied.……..……..……..……..………………  6. Not satisfied at all…………………………….............. | |  |
| Q416 | What is your level of satisfaction concerning the information provided by your GP regarding your health problem?  ***(Choose only one option)*** | 1. Never have been to GP in UK …………...…….….…...  2. Fully satisfied …..……..…..…..….................................  3. Considerably satisfied...……..……..……..……………  4. Moderately satisfied ….....................................................  5. A little satisfied.……..……..……..……..………………  6. Not satisfied at all…………………………….............. | |  |
| Q417 | Could you communicate well with the GP?  ***(Choose only one option)*** | 1. Never had had a health problem/never been to GP….…  2. Could communicate well....…..…..…............................  3. Couldn’t communicate well.…...……………………….. | |  |
| Q418 | Do you want to use interpreter services during your visit to a GP?  ***(Choose only one option)*** | 1. Want to use interpreter service....…..…..…....................  2. Don’t want to use interpreter service…...……………… | |  |
| Q419 | Did you use interpreter services during your last GP consultation?  ***(Choose only one option)*** | 1. Never had had a health problem/never been to GP….…  2. Used interpreter service....…..…..…............................  3. Didn’t use interpreter service…...……………………….. | |  |
| Q420 | Have you been offered interpreter service during your visit to GP?  ***(Choose only one option)*** | 1. Never had had a health problem/never been to GP….…  2. Have been offered interpreter service....…..........................  3. Haven’t been offered interpreter service …………….. | |  |
| Q421 | Did you do any of these during your last illness in UK?  ***(You can choose more than one option if these apply)*** | 1. Never been ill in the UK…………...……………….....  2. Consulted with a Burmese doctor (registered in UK) ……………………………...............................................  3. Consulted with a Burmese doctor (not registered in UK) ………………………………………………….......  4. Bought and took medicine from pharmacy (Boots or other) …………………………………………………….  5. Took traditional medicine brought from Burma ...…...  6. Took western medicine brought from Burma………...  7. Wait for spontaneous recovery………………......…....  8. Went for consultation at GP surgery………………….  9. Others ………………………………………………...  Please specify ____________________________________________  _____________________________________________ |  | |
| Q422 | Were you refused for the treatment by GP during your last illness? | 1. Didn’t go to GP ………………………………….….…  2. Have been refused………………….....…..........................  3. Haven’t been refused …………………………………..  If you have been refused, please give the reason: ____________________________________________________________________________________________________________________________________ | |  |
| Q423 | Please choose the reason which deterred/deterring you from going to GP.  ***(You can choose more than one option if these apply)*** | 1. Never have had health problem in the UK…………….  2. Not registered with a GP………………………………  3. Don’t know how to take the appointment……………  4. Afraid to speak English………………………………..  5. Don’t like western medicine …………………………  6. Feeling the health care in UK has too high for me.…..  7. Feeling that I deserve the suffering because of my previous practice ………………………………………..  8. Afraid of discrimination for being a foreigner …..…..  9. Appointment takes very long…………………………. 10. Due to travel cost ……………………………..……..  11. GP is too far to go………………………...………….  12. Due to time constraint………………………………  13. Went directly to hospital A&E/ walk in centre………  14. Took medicine by myself ……………………………  15. GP surgery has bad hospitality……………………….  16. Waiting time is too long ……………………………..  17. No reason was deterring me to go to GP …….…….. 18. Others ……………………………………………….. (please specify)________________________________ _____________________________________________ |  | |
| Q424 | Do you have any chronic health problem? | 1. Yes ……………………………………………….….…  2. No……………………….....…..…..…............................ | |  |
| Q425 | Are you taking any regular consultation for your chronic health problem? | 1. Do not have chronic health problem …………….….…  2. Taking regular treatment.....…..…..…............................  3. Not taking regular treatment….....…………………….. | |  |
| Q426 | How fluent do you think you can describe your illness in English if you are ill? | 1. Very well …………..……..……..……..……..……..…  2. Well ………......……..……..……..……..……..........…  3. Fair ……………...……..……..……..……..……..……  4. Poor…….………...……...……...……...……...……...  5. Very poor………...……...……...……...……...……... | |  |

**This is the end of the questionnaire. Thank you very much for taking time to answer the question.**
