# Supplementary material for: Access to and utilisation of GP services among Burmese migrants in London: a cross-sectional descriptive study
Source: BMC Health Serv Res. 2010 Oct 12;10:285. doi: 10.1186/1472-6963-10-285 (PMC2970605; doi:10.1186/1472-6963-10-285)
Supplement: Additional file 2 — Pre-survey in-depth interview record sheet. [file 1472-6963-10-285-S2.DOCX]

# Pre-survey IDI record Sheet

|  |  | **Pre-survey IDI data collection sheet** | | |  |
| --- | --- | --- | --- | --- | --- |
| Date: _________________________ | | |  | Name: ________________________ | |
| Occupation: ___________________ | | |  | Duration of Stay: ________________ | |
| **Population mapping** | |  |  |  |  |
|  |  |  |  |  |  |
| **Sr:** | **Area** | **type of community** | **Population estimate** | **Possible contact person** | **Ph:** |
|  |  |  |  |  |  |
|  |  |  |  |  |  |
|  |  |  |  |  |  |
|  |  |  |  |  |  |
|  |  |  |  |  |  |
|  |  |  |  |  |  |
|  |  |  |  |  |  |
|  |  |  |  |  |  |
|  |  |  |  |  |  |
|  |  |  |  |  |  |
|  |  |  |  |  |  |
|  |  |  |  |  |  |
|  |  |  |  |  |  |
|  |  |  |  |  |  |
|  |  |  |  |  |  |
|  |  |  |  |  |  |
|  |  |  |  |  |  |
|  |  |  |  |  |  |
|  |  |  |  |  |  |
|  |  |  |  |  |  |
|  |  |  |  |  |  |
|  |  |  |  |  |  |
|  |  |  |  |  |  |
|  |  |  |  |  |  |
|  |  |  |  |  |  |
|  |  |  |  |  |  |
|  |  |  |  |  |  |
| **Comments on questionnaire** | | |  |  |  |
| …………………………………………………………………………………………………………………………………………………………………... | | | | | |
| …………………………………………………………………………………………………………………………………………………………………... | | | | | |
| …………………………………………………………………………………………………………………………………………………………………... | | | | | |
| …………………………………………………………………………………………………………………………………………………………………... | | | | | |
| …………………………………………………………………………………………………………………………………………………………………... | | | | | |
