# Supplementary material for: Access to and utilisation of GP services among Burmese migrants in London: a cross-sectional descriptive study
Source: BMC Health Serv Res. 2010 Oct 12;10:285. doi: 10.1186/1472-6963-10-285 (PMC2970605; doi:10.1186/1472-6963-10-285)
Supplement: Additional file 3 — Post-survey in-depth interview guideline. [file 1472-6963-10-285-S3.DOCX]

# Post-survey In-depth Interview Guideline

- Introduce interviewer and the project. Ask if interview can be recorded
- Explain that all responses are confidential and no personally identifiable information would be used when writing the thesis
- Mention that the interview may take 30 to 60 minutes

Duration of stay in UK ……………… Number of areas you have lived in UK ……………..

Que 1.1: Could you please tell me about your experience on last illness in London?

Que 1.2: Could you please tell me about your experience on registration in London? How did you do and what did you need? Any reason hindering your GP registration?

Que 1.3: Could you please share me about your experience on last GP visit in London? How was it and what did you need? And reason hindering your GP consultation.

Que 1.4: How do you think the effect of following factors on your illness, GP registration and GP visit? Explore one after another:

- Age & Sex
- Ethnicity and religion
- Language proficiency
- Supporter for GP registration and visit
- Income & Working hour
- Foreign experience (Duration of stay in UK, have been to other foreign countries)
- Visa status
- Knowledge on right and payment regarding the service utilisation

**If time allow and/or there was no significant experience, ask the experience of a friend/family**

Que 2.1: Could you please tell me a significant illness of your friends or family in London?

Que 2.2: Could you please tell me about a significant event regarding GP registration among your friends or family in London? And reason hindering your GP registration for Burmese?

Que 2.3: Could you please share me about a significant event regarding GP visit among your friends or family in London? And reason hindering your GP consultation for Burmese?

Que 2.4: How do you think the effect of following factors on illness, GP registration and GP visit of Burmese in London? Explore one after another:

- Age & Sex
- Ethnicity and religion
- Language proficiency
- Supporter for GP registration and visit
- Income & Working hour
- Foreign experience (Duration of stay in UK, have been to other foreign countries)
- Visa status
- Knowledge on right and payment regarding the service utilisation
