# Supplementary material for: Access to and utilisation of GP services among Burmese migrants in London: a cross-sectional descriptive study
Source: BMC Health Serv Res. 2010 Oct 12;10:285. doi: 10.1186/1472-6963-10-285 (PMC2970605; doi:10.1186/1472-6963-10-285)
Supplement: Additional file 4 — Information sheet for questionnaire survey. [file 1472-6963-10-285-S4.DOCX]

# Information Sheet for Questionnaire Survey

**Questionnaire Survey on**

**Knowledge on and Experience of Primary Health Care among**

**Burmese Migrants in the UK (LONDON)**

The objective of this questionnaire is to ***evaluate the knowledge on and experience of primary health care utilisation among age 15 and above Burmese migrants in London***. Currently the level of Burmese’s health care utilisation and health needs are yet to know. So, needs among the Burmese migrants would be investigated by using the findings obtained from this questionnaire. According to the information received from the survey, needs of Burmese migrants’ health access will be identified which will be utilized to improve their health access in cooperation with relevant organisations.

Hence, if your age is 15 and above and you are/have been a Burmese citizen, we would like to request ***your contribution to help increase the health care status of Burmese abroad*** by participating in this survey. However, the participation in the survey is completely voluntary.

This survey is being conducted by Nyein Chan Aung (a student investigator) with supervision from Dr Bernd Rechel, European Centre on Health of Societies in Transition, London School of Hygiene & Tropical Medicine and Dr. Peter Odermatt, Department of Public Health and Epidemiology, the Swiss Tropical Institute, Basel. This study will form part of his thesis for the Masters of International Health, and has been approved by the Ethical Committee of the London School of Hygiene and Tropical Medicine.

This questionnaire is investigating the factors related to Burmese Migrants’ Access to Primary Health Care. In this questionnaire, you will be asked about the following issues:

- Your background information, immigration status and overseas experience
- Your knowledge on right and payment to access health care for migrants in England
- Your experience of current health care utilisation

No information concerning your personal identification will be asked and as a result ***no one will know what you have answered***. When we write the thesis, everything we find out from this study will be summarised. This also ensures ***no link in the information you are providing with your identity***.

We estimated that the questionnaire will take about 10-15 minutes to complete. ***When you have finished answering the questionnaire, please put it in the stamped envelope provided and post it back.***

If you want any further information, or have any questions about the research, please do not hesitate to contact the student investigator with the following address and mobile number:

Nyein Chan Aung

Ph: 07529725133

c/o Dr Bernd Rechel
European Centre on Health of Societies in Transition
London School of Hygiene & Tropical Medicine
Keppel Street, Room LG 20
London WC1E 7HT

Thank you for your time.

Sincerely,

Nyein Chan Aung (Student Investigator)
